# Dual tasking as a predictor of falls in post-stroke: Walking While Talking versus Stops Walking While Talking.

**DOI:** 10.12688/f1000research.158764.1

**Published:** 2024-11-20

**Authors:** Disha Lamba, Abraham M. Joshua, Vijaya kumar K, Akshatha Nayak, Prasanna Mithra, Rohit Pai, Shivananda Pai, Shyam Krishnan K., Vijayakumar Palaniswamy

**Affiliations:** 1 Department of Physiotherapy, Kasturba Medical College Mangalore, Manipal Academy of Higher Education, Karnataka, Manipal, 576 104, India; 2 Department of Community Medicine, Kasturba Medical College Mangalore, Manipal Academy of Higher Education, Manipal, 576 104, India; 3 Department of Neurology, Kasturba Medical College Mangalore, Manipal Academy of Higher Education, Karnataka, Manipal, 576 104, India

**Keywords:** focus of attention, cognition, stroke, walking, predictive value of tests, risk assessment, ROC curve, executive function.

## Abstract

**Background:**

Falls affect 40-70% within the first year and contributing to increased morbidity and reduced quality of life. Dual-task assessments, such as the Walking While Talking (WWT) and Stops Walking While Talking (SWWT) tests, are potential tools for predicting fall risk, but their comparative effectiveness remains underexplored.

**Methods:**

This cross-sectional study included 68 stroke survivors who completed WWT-Simple (WWT-S), WWT-Complex (WWT-C), and SWWT assessments, as well as the Berg Balance Scale (BBS) and Falls Efficacy Scale (FES). Spearman correlations assessed relationships between balance, fear of falling, and dual-task performance. Logistic regression identified predictors of fall risk, and Receiver Operating Characteristic (ROC) analysis evaluated predictive accuracy. The study adhered to the Strengthening the Reporting of Observational Studies in Epidemiology (STROBE) checklist.

**Results:**

BBS scores were strongly negatively correlated with WWT-S (r = -0.734, p < 0.0001) and WWT-C (r = -0.737, p < 0.0001), indicating poorer balance with slower dual-task completion. Positive correlations were found between WWT-S and FES (r = 0.668, p < 0.0001) and WWT-C and FES (r = 0.610, p < 0.0001), linking slower completion times with higher fear of falling. SWWT was significantly negatively correlated with BBS (r = -0.625, p < 0.0001). WWT tests had higher sensitivity (97.8%) and specificity (99%) than SWWT (sensitivity = 68.9%; specificity = 91.3%). Logistic regression identified SWWT (Positive) as a significant predictor of fall risk (p = 0.009), and ROC analysis showed an AUC of 0.911, indicating excellent predictive power.

**Conclusions:**

Findings highlight the superior predictive value of WWT tests over SWWT in assessing fall risk among stroke survivors. Incorporating dual-task measures into clinical practice may enhance fall risk evaluation, supporting targeted stroke rehabilitation.

## Introduction

Falls after stroke are recognized as a serious public health challenge worldwide, contributing to increased morbidity and reduced quality of life among stroke survivors. ^1, 2^ Falls are quite common in this population due to increased cognitive-motor interference (CMI). This interference impacts their ability to effectively integrate balance, walking, and cognitive tasks. ^3^ Cognitive-motor interference in stroke patients is often characterized by deficits in executive functions, which include challenges with memory, attention, and motor control. ^1, 4, 5^ Notably, difficulties in performing dual tasks—an essential aspect of executive function—are significant risk factors for falls among stroke survivors. ^6– 8^

Impaired postural balance and low falls efficacy are critical factors that contribute to frequent falls in stroke survivors. ^9, 10^ Recent studies, such as those by Xu (2018) ^11^ and Sjöholm et al. (2022), ^1^ highlight the connection between reduced balance and an increased risk of falling. There is growing interest in assessing cognitive-motor interference through dual-task paradigms; however, most previous observational research has primarily relied on established tools like the Berg Balance Scale (BBS) and the Tinetti Falls Efficacy Scale (FES) to evaluate balance and fear of falling. Evidence regarding the predictive ability of dual-task performance tests for fall risk in stroke survivors remains limited.

Dual-task assessments are shown to be reliable for measuring cognitive-motor performance in stroke survivors, ^12^ yet the comparative effectiveness of different dual-task tests in predicting fall risk is underexplored. Studies by Hofheinz and Mibs (2016) ^13^ have validated dual-task tests, such as the Timed Up and Go Test, as effective predictors of fall risk in older adults. However, further research is needed to see if these findings apply to stroke survivors, who have unique cognitive and motor challenges. Emerging research supports the utility of dual-task paradigms like the WWT and SWWT for assessing CMI and identifying fallers. ^4, 7^ These assessments engage higher executive functions that extend beyond automatic walking tasks. ^14, 15^ However, a recent predictive analytics study by Abdollahi et al. (2024) highlighted limitations in current dual-task assessments for fall risk prediction, emphasizing the need for more tailored methods. ^16^

While the clinical value of dual-task assessments in identifying fall risk is established, there remains a paucity of research examining how stroke survivors perform on both WWT and SWWT tests and how these outcomes correlate with fall efficacy (FES) and balance (BBS). Assessing dual-task performance alongside established scales may provide deeper insight into fall mechanisms among stroke survivors and facilitate the development of targeted cognitive or behavioral interventions. ^17^

Therefore, the objective of this study is to determine whether dual-task performance, specifically the Walking While Talking (WWT) and Stops Walking While Talking (SWWT) tests, is correlated with established fall risk predictors such as the Berg Balance Scale (BBS) and the Falls Efficacy Scale (FES). Additionally, the study aims to compare the predictive ability of WWT and SWWT in identifying fall risk among stroke survivors using these standardized fall prediction tools. Finally, the research seeks to identify which of the dual-task assessments, WWT or SWWT, serves as a superior predictor of fall risk in this population.

We hypothesize that stroke survivors with poor dual-task performance on the Walking While Talking (WWT) and Stops Walking While Talking (SWWT) tests will exhibit significant associations with fall risk, as evaluated by the Berg Balance Scale (BBS) and the Falls Efficacy Scale (FES) scores. Additionally, we anticipate that there will be a significant difference in the predictive accuracy between WWT and SWWT in determining fall risk among stroke survivors.

## Methods

### Study setting and design

A cross-sectional study was conducted to compare dual-task performance between Walking While Talking (WWT) and Stops Walking While Talking (SWWT) in predicting fall risk, as assessed by the Berg Balance Scale (BBS) and the Falls Efficacy Scale (FES). The study adhered to the Declaration of Helsinki (World Medical Association) and was reported in accordance with the Strengthening the Reporting of Observational Studies in Epidemiology (STROBE) guidelines. ^18^

### Participants

**Figure 1.**
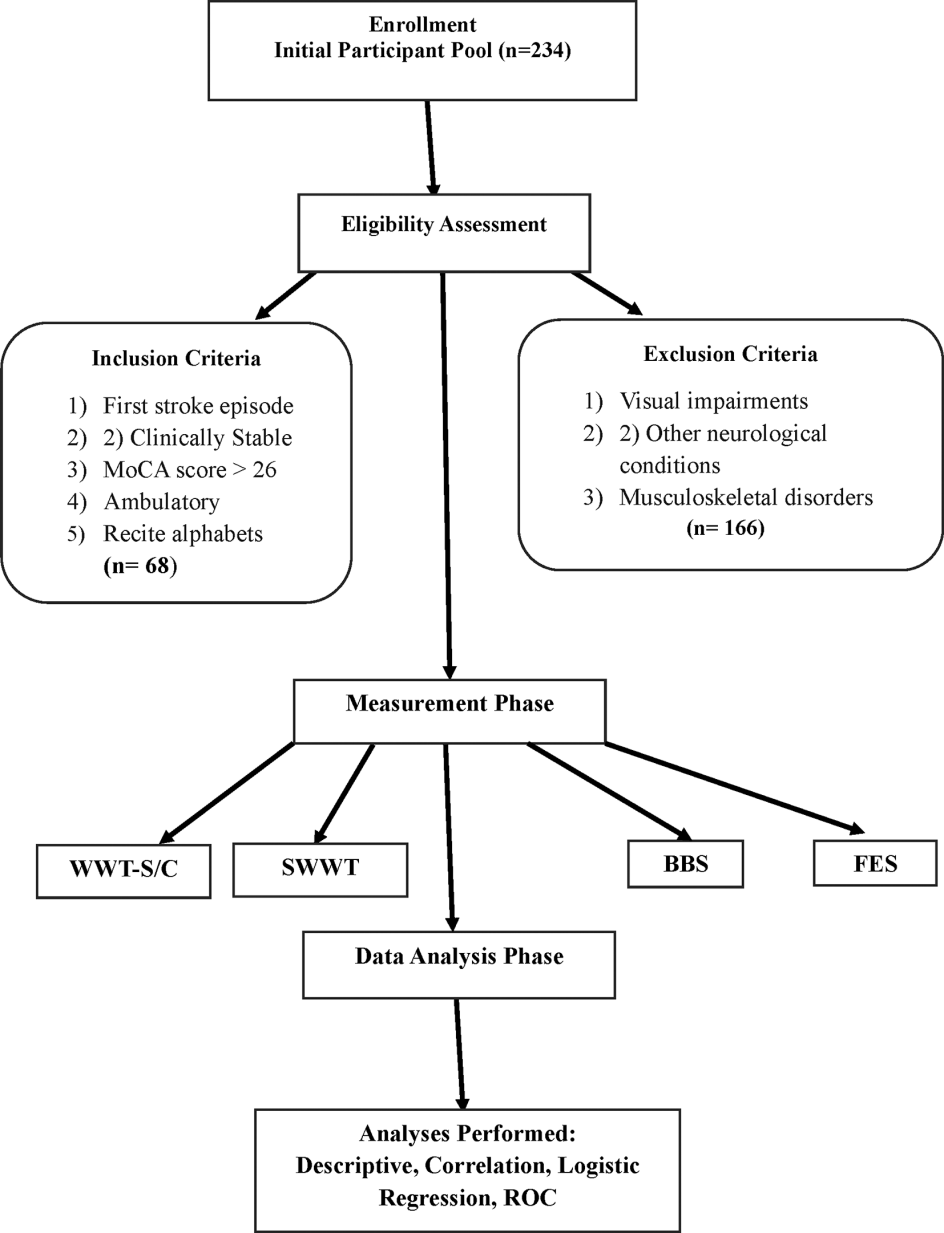
Study flow chart illustrates recruitment, inclusion, and analysis procedures.

A total of 234 potential participants were screened for eligibility ( Figure 1), resulting in a final sample of 68 stroke survivors admitted to Kasturba Medical College Hospital (KMCH), Mangalore, affiliated with Manipal Academy of Higher Education (MAHE), Manipal, from March 2021 to March 2022. All participants received a stroke diagnosis from an experienced neurologist affiliated with KMCH. To mitigate selection bias, the study specifically approached individuals diagnosed with stroke by the neurologist and included only those who met predefined inclusion and exclusion criteria. The purpose of the study was clearly explained to eligible participants, and informed consent was obtained from all individuals prior to their participation.

The inclusion criteria for the study required participants to have experienced a first episode of stroke, maintain a clinically stable status, achieve a Montreal Cognitive Assessment (MoCA) (A official written permission to use MoCA for research purpose has been granted in 2020) score of 26 or higher, demonstrate ambulatory ability, and be able to recite the alphabet in English or their native language. Exclusion criteria included visual impairments, other neurological conditions, and musculoskeletal disorders affecting gait. This selection process aimed to create a homogeneous study population and enhance the reliability of findings related to cognitive-motor interference and fall risk among stroke survivors.

### Data collection, measurement, variables

Data collection for the study involved a standardized single evaluation session for all participants. A qualified and trained postgraduate student in Neurological Physiotherapy, under the supervision and guidance of the research team members (AN and SK), collected demographic data and scores for the Montreal Cognitive Assessment (MoCA) (We have obtained the official written permisison from the MoCA Copyright owners.), Berg Balance Scale (BBS), Falls Efficacy Scale (FES), Walking While Talking (WWT), and Stops Walking While Talking (SWWT). To minimize order effects, participants were randomly assigned to perform either the WWT or SWWT task first ( Table 1).

**Table 1.** Demographic and clinical characteristics of participants (N = 68).

Characteristic	Median (IQR) or N (%)
Age (years)	63 (55.75-71)
**Gender**	
- Male	48 (70.6%)
- Female	20 (29.4%)
**Type of Stroke**	
- Ischaemic	59 (86.8%)
- Haemorrhagic	9 (13.2%)
**Affected Side**	
- Left	38 (55.9%)
- Right	30 (44.1%)
Duration Since Stroke (days)	5 (3-9)
MOCA Score	27 (26-28)
BBS Score	36 (32.25-43.5)
FES Score	39 (21.5-48.25)
WWT-S (sec)	35 (28.75-43)
WWT-C (sec)	44 (34.75-56.25)
SWWT (Positive)	33 (48.5%)
SWWT (Negative)	35 (51.5%)

MOCA - Montreal Cognitive Assessment; BBS - Berg Balance Scale; FES - Falls Efficacy Scale; WWT-S - Walking While Talking Test (Simple); WWT-C - Walking While Talking Test (Complex); SWWT - Stops Walking While Talking (Positive indicates stopping while talking; Negative indicates continuation).

### Screening for cognitive impairment

Cognitive function was screened using the 30-point MoCA test. The MoCA evaluates seven cognitive domains: visuospatial/executive function, naming, attention, language, abstraction, delayed recall, and orientation. ^19^ A MoCA score of 26 or higher served as the cutoff for inclusion. ^19^

### Dependant variable-fall risk assessment

For fall risk assessment, participants underwent evaluations using the BBS and FES. The BBS is a 14-item scale designed to assess static balance and predict fall risk. It has demonstrated high test-retest reliability (ICC = 0.98) ^20, 21^and has been validated in the stroke population. ^22, 23^ The scale ranges from 0 to 56, with lower scores indicating a higher risk of falling. ^24, 25^ The FES is a patient-reported measure that assesses confidence in performing ten daily activities without fear of falling. Higher scores indicate lower confidence, and the FES has shown excellent test-retest reliability (ICC = 0.97). ^26, 27^

### Independent variables-dual-task performance

Participants completed both WWT and SWWT tests as independent variables. The WWT measures the time taken to walk a 20-foot distance while simultaneously reciting the alphabet. Two versions were used. The simple version requires the participant to recite the alphabet in sequence, while the complex version involves reciting every alternate letter (e.g., A-C-E). Times exceeding 20 seconds for the simple version (WWT-S) or 33 seconds for the complex version (WWT-C) indicate a fall risk. The SWWT evaluates participants’ ability to walk and talk simultaneously. Participants were asked to walk while engaging in a conversation; stopping during this task was noted as a higher fall risk indicator.

### Statistical analysis

All statistical analysis was conducted using IBM SPSS Statistics for Windows, Version 25.0. Continuous variables were summarized using medians and interquartile ranges (IQRs) due to non-normal data distribution, confirmed by Shapiro-Wilk tests (p < 0.05).

Group comparisons of BBS and FES scores based on SWWT outcomes (positive vs. negative) were conducted using the Wilcoxon rank-sum test, with W-statistics and p-values reported. The significance level was set at p < 0.001 for these comparisons. Additionally, Spearman’s rank correlation coefficients were calculated to evaluate association between dual-task performance and fall risk measures (BBS and FES). Logistic regression analysis was performed to assess the predictive power of WWT and SWWT, with results presented as model coefficients and p-values.

For analysis of model discrimination, ROC curve analysis was conducted, reporting the area under the curve (AUC) to determine model discrimination. Sensitivity and specificity for each dual-task test were evaluated to assess clinical utility. Statistical significance was set at p < 0.05 unless stated otherwise for specific tests.

## Results

### Participants

A total of 234 stroke participants were screened, of which 68 ambulant participants who met the inclusion and exclusion criteria were included in the study. The mean age of the participants was found to be 63 years. Table 1 presents the demographic and clinical characteristics of the sample. The cohort was predominantly male (70.6%), with the majority (86.8%) having experienced ischemic strokes. In terms of laterality, 55.9% of participants had their left side affected. The median duration since stroke onset was 5 days (IQR: 3-9 days), indicating that the participants were in the early subacute phase of recovery.

Functional assessments revealed that the median Montreal Cognitive Assessment (MoCA) score was 27 (IQR: 26-28), indicating generally preserved cognitive function among participants. The median Berg Balance Scale (BBS) score was 36 (IQR: 32.25-43.5), suggesting a moderate risk of falls, while the median Falls Efficacy Scale (FES) score was 39 (IQR: 21.5-48.25), reflecting varying levels of confidence regarding fall-related concerns. The dual-task performance metrics indicated that the median time for the Walking While Talking Test—Simple (WWT-S) was 35 seconds (IQR: 28.75-43), and for the Walking While Talking Test—Complex (WWT-C), it was 44 seconds (IQR: 34.75-56.25). In the Stops Walking While Talking (SWWT) test, 48.5% of participants exhibited a positive outcome by stopping while talking, indicating difficulties in dual tasking ( Table 1).

**Figure 2.**
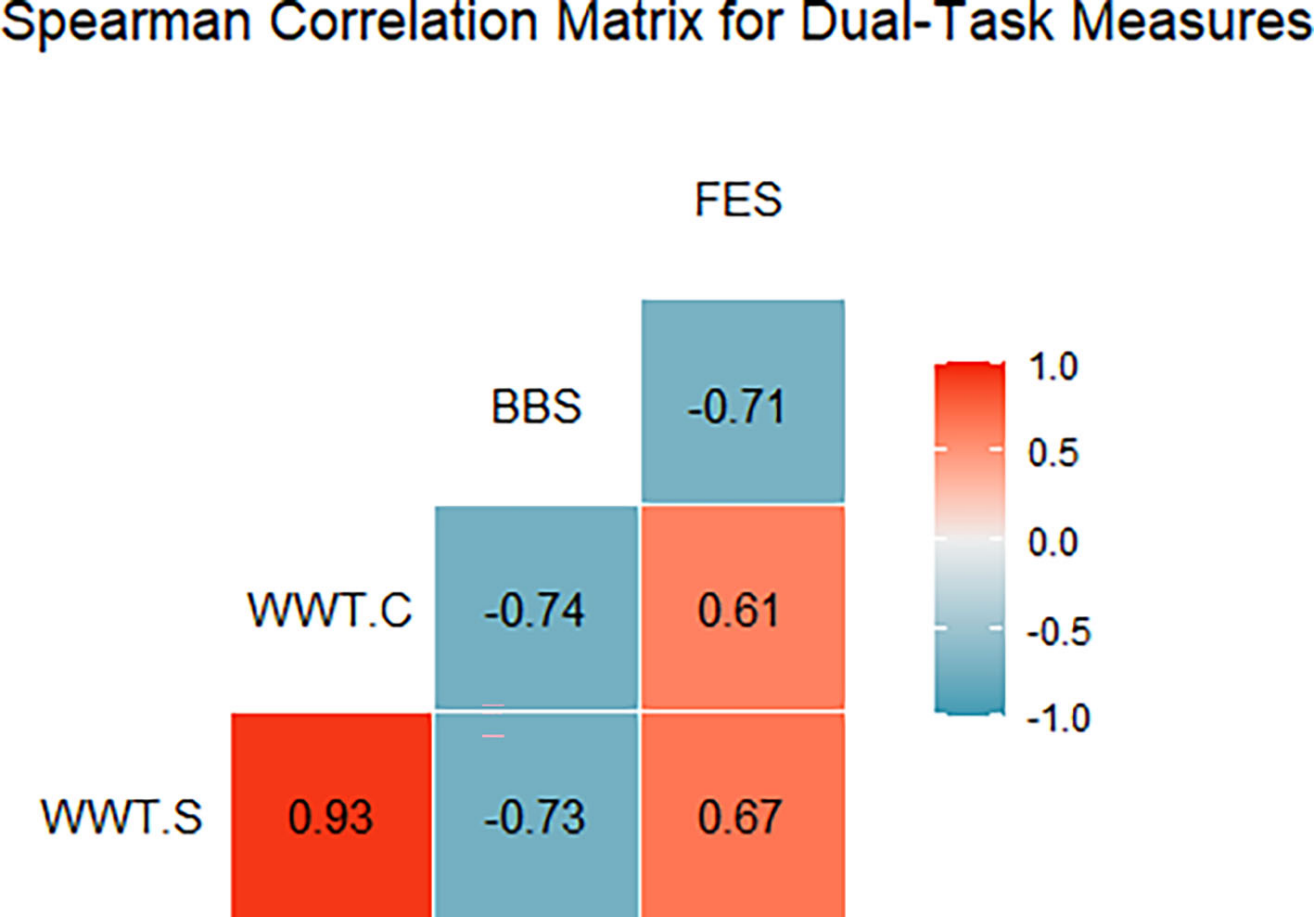
WWT-S (Walking While Talking - Simple), WWT-C (Walking While Talking - Complex), BBS (Berg Balance Scale), and FES (Falls Efficacy Scale). Positive correlations are red, negative are blue, with color intensity showing strength; boxes show coefficients.

Figure 2 illustrates the Spearman correlation matrix for the dual-task performance measures (WWT-S, WWT-C) and conventional fall prediction scales (BBS, FES). The analysis revealed a strong negative correlation between WWT-S and BBS (r = -0.73), indicating that longer times on the simple walking-while-talking test were associated with poorer balance scores. A similar strong negative correlation was found between WWT-C and BBS (r = -0.734) ( Table 2), reinforcing that prolonged times in the complex dual-task test were linked to lower balance capabilities. Additionally, a moderate positive correlation was observed between WWT-S and FES (r = 0.67), suggesting that slower times correlated with a higher fear of falling. A strong negative correlation between BBS and FES (r = -0.71) indicated that lower balance scores were associated with greater anxiety about falling. These findings underscore the importance of dual-task assessments (WWT-S, WWT-C, SWWT) as valuable indicators of fall risk and balance confidence among stroke survivors ( Table 2).

**Table 2.** Correlation of WWT-simple, WWT-complex, and SWWT with BBS.

	BBS	
Variables	r value	*p* value
WWT-simple	-0.734	<0.0001
WWT-complex	-0.737	<0.0001
SWWT	-0.625	<0.0001

Spearman’s Rank Correlation Coefficient; WWT - Walking While Talking test; SWWT - Stops Walking While Talking test; BBS - Berg Balance Scale.

Table 3 summarizes the logistic regression analysis conducted to evaluate the predictive power of dual-task performance measures for fall risk among stroke survivors. The model included the Walking While Talking Test - Simple (WWT-S), Walking While Talking Test - Complex (WWT-C), and the Stops Walking While Talking (SWWT) test as independent variables, with fall risk defined by a Berg Balance Scale (BBS) score of less than 40 as the outcome. The analysis aimed to determine how effectively these dual-task performance measures could predict fall risk in this population, highlighting their potential utility in clinical assessments and interventions for stroke survivors.

**Table 3.** Logistic regression results for fall risk predictors and predictive performance (sensitivity and specificity) for fall risk predictors.

Variable	Estimate	Std. Error	z-value	*p*-value	Sensitivity (%)	Specificity (%)
Intercept	-3.858	1.276	-3.025	0.002		
WWT-S	0.119	0.064	1.879	0.060	97.8	99
WWT-C	-0.008	0.036	-0.228	0.820	97.8	99
SWWT (Positive)	2.322	0.884	2.627	0.009	68.9	91.3

WWT-S: Walking While Talking Test (Simple); WWT-C: Walking While Talking Test (Complex); SWWT: Stops Walking While Talking (Positive indicates stopping while talking).

The analysis indicated that the intercept was statistically significant (Estimate = -3.858, *p* = 0.002), suggesting a baseline probability of fall risk when all predictors are held constant. The Walking While Talking Test - Simple (WWT-S) variable had an estimate of 0.119 ( *p* = 0.060), indicating a positive association with fall risk; however, this association was marginally non-significant at the 0.05 level. This suggests potential relevance that may become significant in larger samples or under different conditions, highlighting the need for further investigation into the relationship between dual-task performance and fall risk among stroke survivors ( Table 3).

Additionally, sensitivity and specificity analyses revealed that the WWT tests (WWT-S and WWT-C) had high sensitivity (97.8%) and specificity (99%) for predicting fall risk, indicating their strong diagnostic accuracy. In contrast, the SWWT test showed lower sensitivity (68.9%) and specificity (91.3%), though it remained a valuable predictor. These findings underscore the clinical value of dual-task assessments, particularly SWWT, in identifying stroke survivors at risk of falls ( Table 3).

The Walking While Talking Test - Complex (WWT-C) variable had an estimate of -0.008 ( *p* = 0.820), indicating a negligible and non-significant relationship with fall risk. In contrast, the Stops Walking While Talking (SWWT) variable demonstrated a significant positive association (Estimate = 2.322, *p* = 0.009), suggesting that participants who stopped walking while talking faced a substantially higher risk of falls. This finding underscores the importance of SWWT as a predictor of fall risk. Overall, the model indicates that while the WWT-S test shows borderline significance, the SWWT outcome is a robust predictor of fall risk among stroke survivors. This highlights the value of dual-task assessments, particularly SWWT, in clinical evaluations of fall risk ( Table 3).

**Figure 3.**
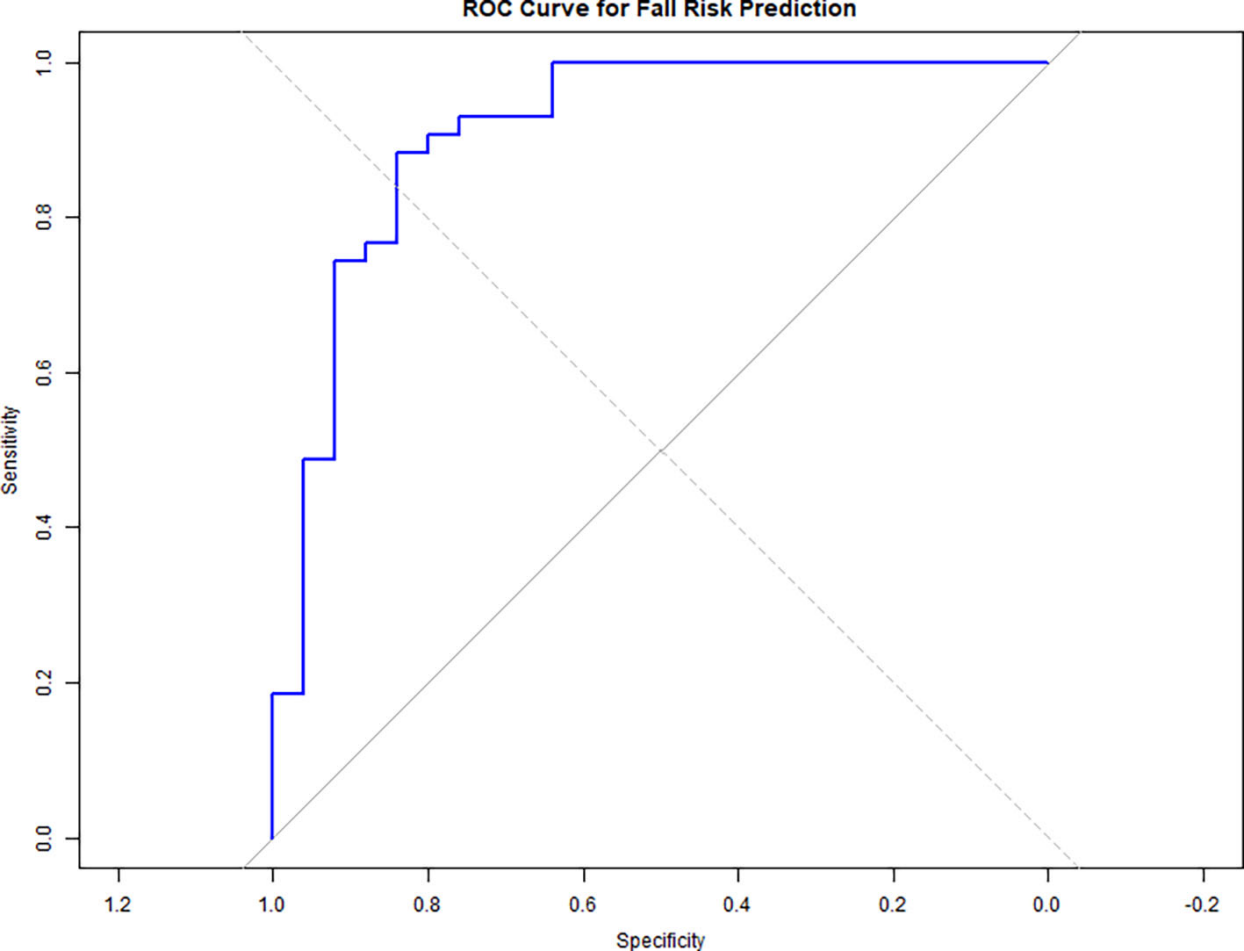
ROC curve shows fall risk prediction accuracy (AUC = 0.911) using dual-task measures (WWT-S, WWT-C, SWWT), indicating excellent discrimination.

A ROC curve analysis ( Figure 3) was conducted to evaluate the predictive accuracy of the logistic regression model for fall risk, which included dual-task performance measures (WWT-S, WWT-C, and SWWT) as predictors. Figure 2 displays the ROC curve, with an Area Under the Curve (AUC) of 0.911, indicating excellent discrimination. This AUC value suggests that the model is highly effective in distinguishing between stroke survivors at risk of falls and those who are not. The ROC curve highlights the utility of dual-task performance assessments in fall risk evaluations. The near-perfect AUC underscores the clinical relevance of incorporating tests like WWT-S, WWT-C, and SWWT into comprehensive fall risk assessments for stroke survivors.

Table 4 shows significant differences in BBS and FES scores between positive and negative SWWT outcomes. Participants who stopped while talking (positive SWWT) had lower BBS scores (W = 994, *p* < 0.001) and higher FES scores (W = 176, *p* < 0.001), indicating poorer balance and greater fear of falling. These results emphasize the link between dual-task performance, balance deficits, and fall-related anxiety in stroke survivors.

**Table 4.** Group comparison of BBS and FES scores by SWWT outcomes.

Outcome measure	SWWT outcome	W-statistic	p-value	Significance
BBS Score	Positive vs. Negative	994	3.198e-07	Significant ( *p* < 0.001)
FES Score	Positive vs. Negative	176	7.955e-07	Significant ( *p* < 0.001)

BBS - Berg Balance Scale; FES - Falls Efficacy Scale; SWWT - Stops Walking While Talking (Positive indicates stopping while talking).

## Discussion

This study aimed to evaluate the predictive ability of dual-task performance measures, specifically the Walking While Talking (WWT) and Stops Walking While Talking (SWWT) tests, in assessing fall risk among stroke survivors. The findings revealed significant associations between dual-task performance and traditional fall risk measures, emphasizing the value of incorporating these assessments into clinical practice for stroke rehabilitation. Previous research has established the importance of dual-task paradigms in identifying fall risk among older adults and patients with neurological impairments. Abdollahi et al. (2024) reported that changes in gait performance under dual-task conditions were significantly associated with fall risk, particularly in frail elderly populations. ^16^ This aligns with our results, where both WWT-S and WWT-C demonstrated strong negative correlations with the Berg Balance Scale (BBS) (r = -0.73 and r = -0.74, respectively), suggesting that slower dual-task completion times indicate poorer balance and an increased risk of falls in stroke survivors. The strong relationship between dual-task performance and balance observed in our study supports the clinical relevance of these assessments.

Conversely, some studies suggest that dual-task assessments offer no significant predictive advantage over single-task evaluations. ^29^ The effectiveness of dual-task assessments may also vary by age group. ^30^ However, the high Area Under the Curve (AUC) observed in our ROC analysis (0.911) for dual-task tests highlights their superior predictive capability. This finding indicates that dual-task assessments, particularly both versions (Simple & Complex) of WWT, offer significant clinical value when used alongside traditional measures like BBS and FES. The high level of discrimination supports their inclusion in comprehensive fall risk assessments for stroke survivors. The high sensitivity (97.8%) and specificity (99%) of the WWT tests show they are very effective at accurately identifying fall risk in stroke survivors. While the SWWT test also has value, its lower sensitivity (68.9%) and specificity (91.3%) make it better suited for quick assessments. These results support using dual-task assessments, especially WWT, in stroke rehabilitation to improve fall prevention strategies.

The SWWT test demonstrated significant predictive power for fall risk (p = 0.009) in our logistic regression analysis, aligning with research advocating for its use as an effective assessment tool. Lundin-Olsson et al. (1997) first highlighted SWWT as valuable for identifying individuals at higher risk of falls in elderly cohorts. ^28^ Our study extends this application to stroke survivors, revealing that participants who stopped walking while talking (SWWT-positive) had a substantially higher risk of falling, reinforcing its utility as an essential part of dual-task evaluation in stroke rehabilitation programs.

The findings supports the clinical value of dual-task performance measures, particularly WWT and SWWT tests, in assessing fall risk among stroke survivors. While previous research has underscored the significance of cognitive-motor interference in fall risk, ^3, 4^ our results enhance this understanding by demonstrating strong correlations with established measures like BBS and FES. This aligns with studies validating dual-task testing as a proxy for evaluating broader functional deficits. ^12^ The predictive power of WWT (both versions), shown by their high sensitivity and specificity, highlights their potential use in comprehensive stroke rehabilitation programs. ^13^

Although our study did not assess gait parameters directly, it is essential to recognize how cognitive-motor interference can affect motor control, contributing to an increased fall risk. ^8^ This is consistent with the understanding that proprioceptive and executive function deficits can influence dual-task performance, impacting balance. ^16^ Incorporating dual-task assessments can provide a more nuanced approach to identifying fall risk beyond traditional single-task methods.

The relationship between falls efficacy, as measured by the Falls Efficacy Scale (FES), and dual-task performance adds an important dimension to understanding fall risk. Our study found moderate positive correlations between WWT-S and FES (r = 0.67) and WWT-C and FES (r = 0.61), indicating that slower completion times were associated with higher fear of falling. These findings align with studies emphasizing the psychological components of balance confidence and their impact on fall risk. Low confidence in balance, reflected in higher FES scores, has been linked to poorer performance in balance and gait tasks.

This study’s relatively small sample size of 68 participants, may limit the statistical power and generalizability of the findings to a broader population. Additionally, the cross-sectional design limits our ability to establish causative relationships between dual-task performance and fall risk over time. Furthermore, conducting the study in clinical settings may not fully represent the real-life challenges that stroke survivors face when multitasking in their daily lives, which could impact the study’s relevance. Finally, while we used the Berg Balance Scale (BBS) and Falls Efficacy Scale (FES) as our main measures—both of which are valid—they might not cover all aspects of fall risk for this group. For future research, it would be beneficial to conduct larger studies over a longer period and to look into additional assessment tools. This could improve our understanding and applicability of findings across different patient groups.

The clinical implications are significant; while the predictive power of SWWT was substantial, the WWT tests demonstrated higher sensitivity and specificity, suggesting they are more effective at accurately identifying individuals at risk. This contrasts with studies finding SWWT to be less sensitive but still valuable for quick assessments. The high sensitivity (97.8%) and specificity (99%) for WWT-S and WWT-C underscore their robustness as reliable indicators for fall risk. Overall, this study contributes to the growing evidence that dual-task performance assessments are crucial for identifying stroke survivors at risk of falls. Acknowledging both significant and non-significant outcomes ensures a balanced discussion that reflects the complexity of dual-task testing in clinical practice. Our findings highlight the superior predictive power of WWT over SWWT, supporting its use in fall risk screening. Future studies should explore longitudinal designs and larger sample sizes to validate these results further and investigate mechanisms underlying dual-task deficits in stroke rehabilitation.

## Author contributions

DL was the principal investigator and enrolled participants for this study. The study was conceptualized by AMJ, AN, and SK. VP conducted the data analysis and led the literature review and critical revision of the manuscript. Supervision of the trial and data collection was carried out by VK, AMJ, and SP, while oversight and guidance during data collection were provided by VP, AN, and SK. RP, VK, and SP made significant contributions to the study design and manuscript preparation. All authors provided critical input and participated in the preparation and final approval of the manuscript.

## Ethics and consent

Approval for the study protocol was obtained on 20/01/2022 from both the Scientific Committee and the Institutional Ethics Committee (Approval #: IEC KMC MLR 01/2022/45) of Kasturba Medical College (KMC) Mangalore, part of the Manipal Academy of Higher Education (MAHE), Manipal. Additionally, the study was registered with The Clinical Trials Registry-India (CTRI/2021/05/033513). The study was conducted in accordance with the ethical principles of the Declaration of Helsinki for research involving human participants Written informed consent was obtained from all participants prior to their inclusion in the study.

## Data Availability

Zenodo: Dual Tasking as a Predictor of Falls in Post-Stroke: Walking While Talking versus Stops Walking While Talking, 10.5281/zenodo.14059154. ^31^ This project contains the following underlying data:
•
Thesis_Data_Sheet_Updated: Excel Database of Raw Data Data are available under the terms of the Creative Commons Attribution 4.0 International license (CC-BY 4.0).
