# Supplementary material for: Dual tasking as a predictor of falls in post-stroke: A cross-sectional analysis comparing Walking While Talking versus Stops Walking While Talking
Source: F1000Res. 2025 Jul 16;13:1395. Originally published 2024 Nov 20. [Version 3] doi: 10.12688/f1000research.158764.3 (PMC12317310; doi:10.12688/f1000research.158764.3)
Supplement: Peer review history

# Reviewer report: Reviewer response for version 3

**DOI:** 10.5256/f1000research.184887.r398748

**Published:** 2025-08-01

**Authors:** Umair Ahmed

**Affiliations:** 1 University Institute of Physical Therapy, University of Lahore, Lahore, Punjab, Pakistan

I have now reviewed the revised version of the manuscript titled “Dual tasking as a predictor of falls in post-stroke: A cross-sectional analysis comparing Walking While Talking versus Stops Walking While Talking” by Lamba et al., along with the authors’ responses to my previous comments.

 The authors have addressed the key concerns raised in my earlier review. Specifically:

 The clarification of the conceptual distinction between Walking While Talking (WWT) and Stops Walking While Talking (SWWT) in the Introduction is appropriate and strengthens the rationale.

 The revised explanation of fall risk assessment (using BBS and FES scores) and the emphasis on associations due to the cross-sectional design are methodologically sound and clearly communicated.

 The justification of the BBS <40 threshold and acknowledgement of limitations, including sample size, absence of power analysis, and lack of internal validation, are appropriate and transparent.

 Corrections to sensitivity and specificity values across the Results section enhance consistency and accuracy.

 The updated Discussion provides a balanced interpretation of WWT-S and SWWT, highlighting their complementary roles in clinical practice rather than suggesting superiority.

 The revised Conclusion accurately reflects the study’s exploratory scope and associative findings.

 Given the revisions and thoughtful responses provided by the authors, I am satisfied with the current version of the manuscript. I now recommend Approval.

 I have no further comments to add.

Is the work clearly and accurately presented and does it cite the current literature?

Partly

If applicable, is the statistical analysis and its interpretation appropriate?

Partly

Are all the source data underlying the results available to ensure full reproducibility?

Partly

Is the study design appropriate and is the work technically sound?

Partly

Are the conclusions drawn adequately supported by the results?

Partly

Are sufficient details of methods and analysis provided to allow replication by others?

Yes

Reviewer Expertise:

Trunk exercises, Fall prediction model, Posture and Balance control.

I confirm that I have read this submission and believe that I have an appropriate level of expertise to confirm that it is of an acceptable scientific standard.

# Reviewer report: Reviewer response for version 2

**DOI:** 10.5256/f1000research.177449.r379756

**Published:** 2025-06-20

**Authors:** Umair Ahmed

**Affiliations:** 1 University Institute of Physical Therapy, University of Lahore, Lahore, Punjab, Pakistan

**Conceptual Framework**

 The study’s rationale is generally well articulated and grounded in existing literature. The authors correctly identify post-stroke falls as a major problem and link it to cognitive-motor interference (CMI) and executive deficits, citing multiple. They note that traditional fall-risk tools (Berg Balance Scale, FES) are widely used but that the predictive value of dual-task tests (Walking-While-Talking [WWT] and Stops-Walking-While-Talking [SWWT]) remains under explored. This provides a clear gap and motivates their comparative study. The objectives and hypotheses follow logically: the authors aim to correlate WWT/SWWT performance with BBS/FES scores and to compare their predictive accuracy for fall risk. Hypotheses are explicitly stated and consistent with the background.

**Strengths**: The introduction is thorough and well-referenced. It cites prior findings (e.g. Abdollahi et al. 2024, Chiaramonte et al. 2022) to justify focusing on WWT and SWWT, and clearly states the study aim and hypothesis. This logical flow is appropriate for a cross-sectional predictive study.

**Suggestions for Improvement**: While the framework is strong, the authors could more explicitly justify the choice of WWT versus SWWT. For example, they mention “higher executive functions” and “cognitive demands” of SWWT, but elaborating why SWWT might be theoretically a better predictor (beyond just empirical testing) would strengthen the rationale. It would also be helpful to clarify that “fall risk” here is defined by balance/fear scores (not actual falls), as this choice underpins their hypotheses. Finally, given the cross-sectional design, the authors might note (even in the introduction) that only associations – not causal effects – can be inferred.

**Statistical Methods**

 The statistical methods are largely appropriate for the design and data. The authors correctly identify non-normal distributions (Shapiro–Wilk, p<0.05) and therefore use medians/IQRs and nonparametric tests. They employ Spearman rank correlation to assess associations among dual-task times and BBS/FES which is suitable for skewed data. For dichotomous “fall risk” (BBS<40), logistic regression is used to identify predictors (WWT-S, WWT-C, SWWT). Finally, ROC analysis is applied to evaluate predictive accuracy (AUC) of the tests. In general, these techniques match the study’s objectives.

 However, several methodological issues deserve comment:

 •    Sample size: With only 68 participants, the logistic model with three predictors is underpowered by some rules of thumb. The authors do not report any a priori power analysis or justify the sample size. A small sample raises concerns about overfitting and unstable estimates (the wide confidence intervals for AUC suggest this). In particular, Table 3’s extremely high sensitivity/specificity for WWT (97.8%/99%) seems implausibly optimistic on a sample of 68. Bootstrapping or cross-validation could have been used to assess overfitting. Future studies should recruit larger cohorts to validate these findings.

 •    Outcome definition: Defining “fall risk” as BBS <40 is unconventional (the BBS is itself a fall-predictor scale). This choice essentially makes the predictors and outcome both balance measures, which may inflate associations. The authors should justify this cut-off or consider actual fall events if possible.

 •    Handling of variables: The methods say all analyses use p<0.05, except Wilcoxon where p<0.001 was used (why stricter for W/W?). No correction for multiple comparisons is discussed, although few tests were done. The authors could also report effect sizes (e.g. odds ratios with CIs from logistic regression) for better interpretation instead of just p-values and regression coefficients.

 •    Non-normal data: The use of nonparametric tests (Spearman, Wilcoxon) is correct. It might also have been informative to transform some variables and check if Pearson or linear regression yields similar results, but this is not essential.

 Suggestions for Improvement: The authors should clarify their strategy for dichotomizing BBS and acknowledge its limitations. They should report odds ratios (or adjusted effect sizes) for logistic predictors. A mention of missing data handling (if any) would be appropriate (none is discussed). It would strengthen the analysis to assess calibration of the logistic model (e.g. Hosmer-Lemeshow test) and to consider internal validation of the ROC/AUC (e.g. bootstrapping). If feasible, alternative regression approaches (e.g. ordinal or linear regression on continuous fall-risk measures) might avoid information loss from dichotomization.

**Results and Discussion**

 Clarity and Accuracy: The results are presented clearly with tables and figures. Table 1 (clinical characteristics) and Table 3 (logistic regression) summarize key data. The authors report Spearman correlations, Wilcoxon tests, logistic coefficients, and AUCs in an organized way. In general, the interpretation matches the values: WWT-S and WWT-C times strongly correlate with BBS (r≈–0.73), and WWT-S correlates moderately with fear (FES r≈+0.67). Logistic regression shows SWWT (stopping while talking) significantly predicts fall-risk (p=0.009), while WWT-S is only marginal (p=0.060) and WWT-C is nonsignificant.

 However, one important inconsistency should be noted: the reported sensitivity/specificity values are contradictory. Table 3 and its caption show WWT-S/C sensitivity 97.8% and specificity 99%, but the text and Figure 3 description state WWT sensitivity/specificity as 70%/75% and SWWT as 80%/78%. It is unclear which numbers are correct or how “WWT” (combined simple and complex?) was defined in the latter statement. This discrepancy undermines the clarity of the findings. The discussion should reconcile these figures: either WWT offers near-perfect sensitivity or only ~70%, which have very different clinical implications.

 In the Discussion, the authors appropriately relate their findings to prior work. They note that both WWT and SWWT show predictive ability, and that SWWT’s higher cognitive demands may explain its edge. They cite Abdollahi (2024) and others to support the link between dual-task performance and balance/fall risk. The authors also acknowledge conflicting literature (some studies find no added benefit of dual tasks) and argue that their high AUC (0.911) demonstrates strong discrimination. Importantly, they list multiple limitations near the end: small sample, cross-sectional design, laboratory settings, task complexity, stroke stage heterogeneity, and use of BBS/FES as imperfect proxies. These caveats are critical. Overall, the discussion is comprehensive.

**Suggestions for Improvement**: Despite its thoroughness, the discussion could be more concise and consistent. For instance, it alternates between praising WWT and SWWT. Early on, SWWT is highlighted as a “robust predictor”, but later the authors conclude that WWT actually had superior sensitivity/specificity and “superior predictive power”. This flip-flop is confusing. The authors should clearly specify which metric (AUC vs. sensitivity) drives each conclusion and avoid contradictory language. It may be more accurate to say each test has strengths (WWT yielded higher sensitivity in their analysis, whereas SWWT showed higher AUC) rather than claiming one is categorically “better.”

 Additionally, some results interpretations are overstated. For example, stating that WWT-S and WWT-C have “97.8% sensitivity” in the same breath as concluding “dual-task tests have excellent predictive power” may overstate confidence given the study’s limitations. The authors should temper such claims by reminding readers of the exploratory, hypothesis-generating nature of the analysis.

 Finally, the presentation of results could be improved by explicitly reporting confidence intervals and effect sizes (odds ratios) in the text or tables. For example, Table 3 could show OR = exp(coefficient) for easier interpretation. This would also help readers gauge the practical significance of, say, a 10% increase in fall-risk odds per second slower WWT time.

**Conclusion**

 The authors conclude that WWT and SWWT are useful fall-risk assessments in post-stroke care, with some suggestion that SWWT may be slightly better (due to cognitive demand) but WWT had higher sensitivity. These conclusions are partially justified by the data, but should be phrased more cautiously. Given the cross-sectional design, the term “predictive” is somewhat imprecise – the analyses show associations with established risk markers (BBS, FES) rather than prospective prediction of actual falls. The authors do acknowledge this limitation, but the conclusion could emphasize it more.

 For example, rather than claiming that WWT/SWWT predict fall risk, a more cautious phrasing might be that poorer performance on these tasks is associated with higher falls risk scores in stroke survivors. The statement that “WWT has superior predictive power” contradicts earlier statements and should be qualified (e.g. “in our sample, WWT tests identified nearly all at-risk individuals, whereas SWWT was more specific”). In sum, the data support recommending inclusion of dual-task tests in assessment, but any assertion of one test’s superiority should be made tentatively.

**Suggestions for Improvement**: The conclusion should explicitly note that these findings are hypothesis-generating due to the cross-sectional, small-sample nature of the study. Rewording to “Our results suggest that WWT and SWWT are promising tools…” would be appropriate. Emphasis on how future longitudinal studies are needed would also be prudent. Ensuring consistency (e.g. whether SWWT or WWT is “better”) and linking back to the initial hypotheses will make the takeaway clearer.

 Overall, this manuscript provides a detailed exploration of dual-task tests in post-stroke fall-risk assessment. With clearer explanation of the statistical findings, unified conclusions, and a cautious tone regarding limitations, the paper would be significantly strengthened.

 While the article presents a clinically relevant and well-articulated exploration of dual-task assessments for fall risk in post-stroke individuals, it requires specific revisions to strengthen its scientific merit:

 Minor but Important Issues:

 1. Outcome Definition: Using BBS < 40 as a binary proxy for fall risk is methodologically questionable, especially since both predictor and outcome are balance-based. The authors should either:o Use BBS as a continuous variable, oro Justify the chosen cut-off with references.

 2. Statistical Reporting:

 o Inconsistent sensitivity/specificity values between Table 3 and Figure 3 need to be reconciled.

 o Effect sizes such as odds ratios with confidence intervals should be reported for clarity.

 o A brief discussion on power limitations due to small sample size should be added.

 3. Interpretation and Conclusion:

 o Some conclusions overstate the predictive power given the cross-sectional design.

 o Language should be more cautious and consistent regarding which test (WWT vs SWWT) is superior.

 4. Model Validation:o ROC and logistic regression findings need clearer validation (e.g., internal validation or bootstrapping) to ensure generalizability. The study is promising and makes a valuable contribution to clinical neurorehabilitation, especially in resource-limited settings. However, without addressing the above reservations, its conclusions may risk overstatement or misinterpretation.

 Once these revisions are made, the article would merit full approval.

Is the work clearly and accurately presented and does it cite the current literature?

Partly

If applicable, is the statistical analysis and its interpretation appropriate?

Partly

Are all the source data underlying the results available to ensure full reproducibility?

Partly

Is the study design appropriate and is the work technically sound?

Partly

Are the conclusions drawn adequately supported by the results?

Partly

Are sufficient details of methods and analysis provided to allow replication by others?

Yes

Reviewer Expertise:

NA

I confirm that I have read this submission and believe that I have an appropriate level of expertise to confirm that it is of an acceptable scientific standard, however I have significant reservations, as outlined above.

# Reviewer report: Reviewer response for version 2

**DOI:** 10.5256/f1000research.177449.r363554

**Published:** 2025-02-08

**Authors:** Rita Chiaramonte

**Affiliations:** 1 University of Catania, Catania, Italy

Dear Authors,

 I appreciate your efforts in improving the manuscript. I think it is clear and interesting for the readers of the journal.

 Best Regards

Is the work clearly and accurately presented and does it cite the current literature?

Partly

If applicable, is the statistical analysis and its interpretation appropriate?

I cannot comment. A qualified statistician is required.

Are all the source data underlying the results available to ensure full reproducibility?

Yes

Is the study design appropriate and is the work technically sound?

Yes

Are the conclusions drawn adequately supported by the results?

Yes

Are sufficient details of methods and analysis provided to allow replication by others?

Yes

Reviewer Expertise:

Rehabilitation

I confirm that I have read this submission and believe that I have an appropriate level of expertise to confirm that it is of an acceptable scientific standard.

# Reviewer report: Reviewer response for version 1

**DOI:** 10.5256/f1000research.174402.r350983

**Published:** 2025-01-03

**Authors:** Rita Chiaramonte

**Affiliations:** 1 University of Catania, Catania, Italy

This interesting study showed as dual tasking can be a predictor of falls in post-stroke.

 Here are my comments.

**TITLE:** I suggest adding the study design in the title.

**KEYWORDS**: I propose to use Mesh keywords to better contextualize future research

**ABSTRACT:**

1. In the background, you started with epidemiologic data, but I suggest avoiding this. Instead, refer to relevant references in the introduction with the correct citation.
2. At the end of the background, explicitly state the aim of the study.
3. In the introduction, you wrote, “Most previous observational research has primarily relied on established tools like the Berg Balance Scale (BBS) and the Tinetti Falls Efficacy Scale (FES) to evaluate balance and fear of falling,” but without references, even though you referred to previous research. I propose adding the necessary references, particularly regarding the correlation of other scales with dual-task performance in stroke patients. In a recent study, the Tinetti gait test was significantly related to TUG, the Tinetti balance test to the Barthel Index, BBS, and TUG. Finally, there is a positive correlation between the use of aids and BBS and the total score of the Tinetti test (Chiaramonte R, et al., 2024 [Ref 1]). You can referred to this.

**METHODS:** Are the patients in the acute or subacute phase of stroke (refer to the days since stroke indicated in Table 1)?

**FIGURE 1:** Add the legend for the acronyms. Also, include the number of patients that remained in the study, in the measurement phase, and in data analysis (and not only in the inclusion criteria).

**DISCUSSION:**

1. At the beginning, avoid showing the aim of the study; focus on the study’s findings.
2. It would be interesting to discuss the most commonly used tests in proprioceptive and Dual-Task Training after stroke. It would be valuable to better contextualize the need to select appropriate assessments for stroke patients both at baseline and after rehabilitation (Chiaramonte R, et al., 2022 [Ref 2]). This is especially important to support your conclusions about future studies that should explore the topic in stroke rehabilitation.

**LIMITATIONS:** At the end of the discussion, add a paragraph addressing the study’s limitations.

Is the work clearly and accurately presented and does it cite the current literature?

Partly

If applicable, is the statistical analysis and its interpretation appropriate?

I cannot comment. A qualified statistician is required.

Are all the source data underlying the results available to ensure full reproducibility?

Yes

Is the study design appropriate and is the work technically sound?

Yes

Are the conclusions drawn adequately supported by the results?

Yes

Are sufficient details of methods and analysis provided to allow replication by others?

Yes

Reviewer Expertise:

Rehabilitation

I confirm that I have read this submission and believe that I have an appropriate level of expertise to confirm that it is of an acceptable scientific standard, however I have significant reservations, as outlined above.

## References

[1] : The Effectiveness of Goal-Oriented Dual Task Proprioceptive Training in Subacute Stroke: A Retrospective Observational Study. *Ann Rehabil Med* .2024;48(1) : 10.5535/arm.23086 31-41 10.5535/arm.23086 38433007 PMC10915301

[2] : Proprioceptive and Dual-Task Training: The Key of Stroke Rehabilitation, A Systematic Review. *J Funct Morphol Kinesiol* .2022;7(3) : 10.3390/jfmk7030053 10.3390/jfmk7030053 35893327 PMC9326539

# Response

**Published:** 2025-01-23

**Authors:** vijayakumar palaniswamy

**Affiliations:** Institute of Physiotherapy, Srinivas University, Mangaluru, Karnataka, India

**Dear Dr. Rita Chiaramonte**, University of Catania, Catania, Italy

 Thank you for taking the time to review our manuscript titled, *“Dual tasking as a predictor of falls in post-stroke: A cross-sectional analysis comparing Walking While Talking versus Stops Walking While Talking.”* We truly appreciate your insightful and constructive feedback, which has helped us refine and improve our work. Below, we have provided a detailed, point-by-point response to each of your comments.

 TITLE

**Reviewer Comment:** I suggest adding the study design in the title.

**Response:** We have revised the title to explicitly include the study design:

*“Dual tasking as a predictor of falls in post-stroke: A cross-sectional analysis comparing Walking While Talking versus Stops Walking While Talking.”*

 KEYWORDS:

**Reviewer Comment:** I propose using MeSH keywords to better contextualize future research.

**Response:** The keywords have been updated to include MeSH terms: “attention,” “cognition disorders,” “stroke rehabilitation,” “walking,” “predictive value of tests,” “risk assessment,” “ROC curve,” and “executive function.” These terms enhance the contextual relevance for future research.

 ABSTRACT:

**Reviewer Comment:** In the background, you started with epidemiologic data, but I suggest avoiding this. Instead, refer to relevant references in the introduction with proper citations.

**Response:** We revised the background section of the abstract to remove epidemiologic data. It now succinctly highlights the significance of dual-task assessments in stroke rehabilitation with appropriate references.

**Reviewer Comment:** At the end of the background, explicitly state the aim of the study.

**Response:** The aim has been explicitly included:

*“This study aimed to evaluate the predictive value of WWT and SWWT tests in relation to established measures of fall risk among stroke survivors.”*

**INTRODUCTION:**

**Reviewer Comment:** Add references for previous observational research on the correlation of scales like the Berg Balance Scale (BBS) and Tinetti Falls Efficacy Scale (FES) with dual-task performance. Consider including findings from recent studies such as Chiaramonte et al., 2024 [Ref 1].

**Response:** The necessary references have been added, and the text now reads:

*“Studies have demonstrated strong correlations between the Tinetti balance test and the Barthel Index, BBS, and TUG, emphasizing their utility in assessing mobility and independence among stroke survivors (Chiaramonte et al., 2024).”*

 METHODS:

**Reviewer Comment:** Are the patients in the acute or subacute phase of stroke?

**Response:** We clarified that participants were in the subacute phase (3 to 11 weeks post-onset), as indicated in Table 1. This is now explicitly stated in the “Participants” subsection.

 FIGURE 1:

**Reviewer Comment:** Add a legend for the acronyms. Include patient numbers at each phase, such as those who remained in the study, in the measurement phase, and in data analysis.

**Response:** Figure 1 has been updated with:

- A legend for acronyms (WWT, SWWT, BBS, FES).
- Patient numbers at each phase:
  - Screened: 234
  - Eligible: 80
  - Completed: 68

**DISCUSSION:**

**Reviewer Comment:** Avoid stating the study’s aim at the beginning. Focus on the findings instead.

**Response:** The discussion now opens with key findings:

*“This study highlights the significant associations between dual-task performance and traditional fall risk measures, emphasizing the value of incorporating these assessments into clinical practice for stroke rehabilitation.”*

**Reviewer Comment:** Discuss the most commonly used tests in proprioceptive and dual-task training after stroke to contextualize the need for selecting appropriate assessments, citing Chiaramonte et al., 2022 [Ref 2].

**Response:** We expanded the discussion to include a comparison of commonly used tests (e.g., BBS, FES, TUG) and their limitations. This section also underscores the value of dual-task assessments at different stages of stroke rehabilitation, citing Chiaramonte et al. (2022).

 LIMITATIONS

**Reviewer Comment:** Add a paragraph addressing the study’s limitations.

**Response:** We already included a paragraph addressing the study’s limitations in the original manuscript. Based on the reviewer’s recommendation, we have expanded this section to include additional points:

- The type and complexity of cognitive tasks during testing may affect performance and fall risk identification.
- Varying stages of stroke recovery may impact dual-task performance and generalizability.

Thank you once again for your thoughtful feedback, which has significantly improved the quality of our manuscript. We hope the revised version meets your expectations and look forward to hearing your thoughts.

 Kind regards,

 Vijayakumar Palaniswamy

 On behalf of all authors
